# Effects of Tea Tree Oil on Production Performance, Serum Parameter Indices, and Immunity in Postpartum Dairy Cows

**DOI:** 10.3390/ani13040682

**Published:** 2023-02-15

**Authors:** Cong Yuan, Xiaoyu Ma, Maocheng Jiang, Tianyu Yang, Miao Lin, Guoqi Zhao, Kang Zhan

**Affiliations:** 1 Institute of Animal Culture Collection and Application, College of Animal Science and Technology, Yangzhou University, Yangzhou 225009, China; 2 Institutes of Agricultural Science and Technology Development, Yangzhou University, Yangzhou 225009, China; 3 Joint International Research Laboratory of Agriculture and Agri-Product Safety, the Ministry of Education of China, Yangzhou University, Yangzhou 225009, China

**Keywords:** tea tree oil, lipid metabolism, immunity, postpartum

## Abstract

**Simple Summary:**

Tea tree oil (TTO) plays an important role in regulating lipid metabolism and has anti-inflammatory properties. However, the effects of TTO in postpartum dairy cows remain to be investigated. The experimental results show that dry matter intake (DMI) tended to increase (*p* = 0.07) in the 0.01% TTO group relative to that in the control group. Remarkably, the levels of globulin (GLO) and immunoglobulin G (IgG) were elevated (*p* < 0.05) in the TMR diet supplemented with 0.02% TTO compared to those in the control. In addition, 0.02% TTO increased (*p* < 0.05) serum glucose levels. In conclusion, these results suggest that TTO could improve lipid metabolism and enhance immunity in postpartum dairy cows. TTO may be a novel resolution strategy for body condition recovery and milk performance improvement.

**Abstract:**

Tea tree oil (TTO) plays an important role in regulating lipid metabolism and has anti-inflammatory properties. In postpartum dairy cows, dry matter intake (DMI) is dramatically decreased, resulting in lipid metabolism disorder and the systemic pro-inflammatory response. However, the effects of TTO on glucolipid metabolism and immunity in postpartum dairy cows remain uninvestigated. Therefore, this study aimed to evaluate the effects of TTO on production performance, serum biochemical indicators, and immunity in postpartum dairy cows. Our results demonstrate that DMI tended to increase (*p* = 0.07) in the total mixed ration (TMR) diets supplemented with 0.01% TTO/dry matter (DM) basis relative to that in the control group. The 4% fat-corrected milk (FCM) content in the 0.01% and 0.02% TTO groups showed an increase (*p* = 0.09) compared with that in the control. Remarkably, the levels of globulin (GLO) and immunoglobulin G (IgG) were elevated (*p* < 0.05) in the TMR diet supplemented with 0.02% TTO compared to those in the control group. The TTO caused no profound changes in cholesterol (CHO), triglyceride (TG), high-density lipoprotein (HDL), or low-density lipoprotein (LDL). Notably, 0.02% TTO increased (*p* < 0.05) the serum glucose concentration relative to that in the control group. In conclusion, our results demonstrate that TTO could improve glucolipid metabolism and enhance immunity in postpartum dairy cows. It may be a novel resolution strategy for body condition recovery and the improvement of milk performance.

## 1. Introduction

The perinatal period refers to the three (3) weeks before the birth of a calf to 3 weeks after the birth [1], which is recognized as the most challenging phase in dairy cows [2]. In postpartum dairy cows, dry matter intake (DMI) is dramatically decreased, resulting in a negative energy balance (NEB) and dramatic changes in physiology [3]. The dysregulated lipid mobilization triggers a massive release of non-esterified fatty acid (NEFA) into the blood, coupled with severe pro-inflammatory responses [4]. In postpartum, dairy cows exhibit low immunity, leading to a series of pro-inflammatory responses, such as mastitis and metritis. Currently, antibiotic treatment is commonly used to kill pathogens and relieve the inflammatory response. However, many consumers are worried about developing resistance to antibiotic agents due to the development of drug resistance in dairy cows and its occurrence in milk, which can be passed on to humans. Therefore, other therapeutic strategies to kill pathogens and promote immunity are urgently needed to safeguard human health and improve milk quality.

Recent studies have focused on the development of natural plant essential oils as feed additives. Many plant extracts and essential oils derived from some plants contain secondary metabolites that show antibacterial activity properties [5,6]. Tea tree oil (TTO), an essential oil extracted from the leaves of *Melaleuca alternifolia* (*M. alternifolia*), plays a vital role in pathogen inhibition and has anti-inflammatory properties [7,8]. TTO is mainly composed of monoterpenes, double monoterpenes, and corresponding alcohols. The main component is terpinen-4-ol, accounting for about 40 % of the total composition, followed by γ-terpinen and α-terpinen, accounting for about 23% and 10% of the total content, respectively, and about 15% of 1,8-cineole [9]. Studies have shown that TTO not only has antibacterial, anti-inflammatory, antioxidant, antiviral, and immune-enhancing effects [9], but that it also has no toxic side effects, and it has been widely used in medical pharmaceuticals, daily chemicals, and food flavors. However, to the best of our knowledge, the effects of TTO on production performance, glucolipid metabolism, and immunity in postpartum dairy cows are yet to be reported. 

We hypothesized that TTO could improve production performance and enhance immunity in postpartum dairy cows because of the effect of TTO on pathogen inhibition and in relieving the pro-inflammatory response. Therefore, the objective of this study was to evaluate the effects of TTO on production performance, serum biochemical indices, and immunity in postpartum dairy cows fed TMR diets supplemented with TTO. This would provide a scientific theoretical basis for applying TTO in dairy cow production.

## 2. Materials and Methods

### 2.1. Preparation of Tea Tree Oil

TTO preliminary products were obtained from Jiangxi Huabang Biotechnology Co., Ltd (Shangrao, China). The TTO was previously examined using a 9790 gas chromatograph fitted with a flame ionization detector in our laboratory, and its main components are terpinen-4-ol, α-terpinene, α-terpineol, α-pinene, p-cymene, 1,8-cineole, γ-Terpinene, terpinolene, and pinocarveol [9].

### 2.2. Animals

This research was conducted in compliance with the principles of Yangzhou University, the Institutional Animal Care and Use Committee (SYXK (Su) IACUC 2012-0029). Twenty-four healthy parturient Holstein cows were sorted into 3 groups: control (without TTO supplementation, *n* = 8), 0.01% (with TTO supplementation, *n* = 8), and 0.02% (with TTO supplementation, *n* = 8) on a dry matter (DM) basis. All cows were free of clinical signs of disease. The dairy cows were housed in a tie-stall barn located at Yangzhou University Dairy Production Research and Teaching Center. The experimental periods were 21 d in total, comprising 0 d of treatment and 21 d of rearing. The dairy cows were fed thrice daily on a total mixed ration (TMR), which satisfied 100% of NRC demands (Table 1), and they had free access to water. The cows were milked thrice at 4:00, 11:00, and 19:00, respectively.

### 2.3. Sample Collection and Index Determination

#### 2.3.1. Feed Intake Sampling and Analysis

Feed intake was recorded weekly before morning feeding. The dairy cows were fed ad libitum to ensure full intake. Each cow’s feeding quantity and residual quantity were measured three times a week. DMI was calculated based on a 10% residual quantity. DMI = (quantity of feed supplied − remaining quantity) × dry matter%. Feed efficiency = 4% FCM/DMI.

#### 2.3.2. Milk Sampling and Analysis

The cows were milked thrice daily at 0400 h, 1100 h, and 1900 h in their stalls. Milk yield was recorded weekly, and 50 mL milk samples were collected once a week during each milking and mixed according to a ratio of 4:3:3. The milk samples were stored with preservative (Bronopol tablet; D&F Control Systems, San Ramon, CA, USA) at 4 °C. The mixed milk samples were analyzed for milk fat using FTIR and milk F A profile using GLC as described by Urrutia and Harvatine [10].

#### 2.3.3. Blood Collection and Sampling Analysis

On the last day of the experimental treatments, 10 mL blood samples were collected from the coccygeal vessel using a vacuum blood collection device after 3 h of feeding. The tubes were left standing for 30 min and then centrifuged at 3000 r/min for 20 min to obtain serum samples, one of which was immediately stored at −80 °C; the other serum sample was analyzed for alanine aminotransferase (ALT), aspartate aminotransferase (AST), alkaline phosphatase (ALP), total protein (TP), albumin (ALB), globulin (GLO), creatinine (CREA), blood urea nitrogen (BUN), glucose, cholesterol (CHO), triglyceride (TG), high-density lipoprotein (HDL), low-density lipoprotein (LDL), and total bilirubin (TBIL). These indices were detected using a biochemical analyzer (Mindray; BS-420) and the kits (BioSino Bio-Technology & Science, Bejing, China) mentioned above.

#### 2.3.4. Elisa Analysis

IgG, immunoglobulin M (IgM), and immunoglobulin A (IgA) were detected using an Elisa kit (Beijing Sino-Uk Institute of Biological Technology, Bejing, China; intra-assay CV ≤ 5%; interassay CV ≤ 10%).

### 2.4. Statistical Analysis

The experimental data followed a normal distribution and were analyzed using the One-Way ANOVA program in SPSS 25.0 (Norman H. Nie, C. Hadlai (Tex) Huli, Dale H. Bent, Standford University, United States). AM. Duncan’s method was used for multiple comparisons, and the results are expressed as means; differences were declared significant at *p* < 0.05 and tendencies at 0.05 < *p* < 0.10.

## 3. Results

### 3.1. Effects of TTO on Production Performance in Postpartum Dairy Cows

**Figure 1:**
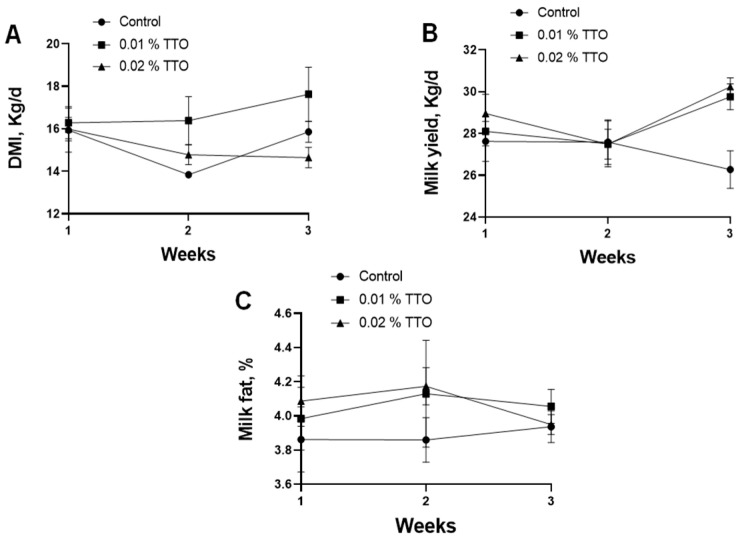
Effects of TTO on DMI, milk yield, and milk fat in different postpartum periods. (**A**) Effects of TTO on DMI in different postpartum periods. (**B**) Effects of TTO on milk yield in different postpartum periods. (**C**) Effects of TTO on milk fat in different postpartum periods.

DMI tended to increase (*p* = 0.07) in the 0.01% TTO group compared to that in the control group (Table 2). The 4% fat-corrected milk (FCM) tended to increase (*p* = 0.09) in both the 0.01% and 0.02% TTO groups. Milk yield and feed efficiency had no profound alterations in the groups treated with TTO compared with those in the control group in the postpartum dairy cows. In addition, DMI, milk yield, and milk fat were not changed by the addition of TTO in the different postpartum periods (Figure 1).

### 3.2. Effects of TTO on Serum Biochemical Indices in Postpartum Dairy Cows

Compared with the control group, the 0.01% and 0.02% TTO groups had no profound effects on the activities of ALT and ALP in the postpartum cows (Table 3). Remarkably, the activity of AST in the serum of the dairy cows tended to decrease (*p* = 0.063) in the 0.02% TTO group relative to that in the control.

### 3.3. Effects of TTO on Serum Nitrogen Content in Postpartum Dairy Cows

Compared with the control group, the 0.01% and 0.02% TTO groups had no profound changes in serum TP, CREA, or BUN in the postpartum dairy cows (Table 4). However, the level of ALB in the serum tended to increase (*p* = 0.08) in the 0.01% TTO group compared with that in the control group. The concentration of GLO increased (*p* < 0.05) in the 0.02% TTO group relative to that in the control group in the postpartum dairy cows.

### 3.4. Effects of TTO on Serum Glucolipid Metabolism in Postpartum Dairy Cows

Compared with the control group, the serum levels of CHO, TG, HDL, and LDL were not changed by the addition of the 0.01% and 0.02% TTO treatments (Table 5). Notably, the glucose concentration increased (*p* < 0.05) in the 0.02% TTO group relative to that in the control group in the postpartum dairy cows.

### 3.5. Effects of TTO on Serum Immune Indices in Postpartum Dairy Cows

The TTO had no profound effects on the serum levels of IgM and IgA in the postpartum dairy cows. Interestingly, the serum concentration of IgG was elevated (*p* = 0.045) in the TMR diets with the 0.02% TTO treatment compared with that in the control group (Table 6).

## 4. Discussion

Previous studies have shown that NEB occurrence in postpartum dairy cows causes body disorders, leading to fatty liver, mastitis, and ketosis [11]. Currently, antibiotic treatment is usually used to kill pathogens and relieve the inflammatory response. However, many consumers are worried due to the development of drug resistance in milk production, which can be passed on to humans. TTO extract is rich in terpine-4-ol, γ-terpene, α-terpene, and 1, 8-cineole, and it has good anti-inflammatory, antioxidant, immune-enhancing, and antibacterial properties and certain effects on improving animal production and preventing diseases [9]. DMI is one of the key factors reflecting dairy cows’ production levels and production performance [12]. An increased DMI can relieve the NEB in postpartum dairy cows. Many factors influence DMI, such as the breed or stage of lactation of the dairy cows, diet, and pasture management.

A previous study showed that TTO can effectively reduce the feed-to-weight ratio of weaned piglets and increase the daily gain of weaned piglets [13]. Our study found that DMI tended to increase with the addition of 0.01% TTO to the TMR diet, indicating that the addition of TTO did not adversely affect the DMI of the dairy cows. Milk fat is the most variable component in milk, and the rest of the constituents change with the milk fat. In the early stage of lactation, milk components are the basis for calves to acquire immunity. Our study found that adding 0.01% and 0.02% TTO to the TMR diet tended to increase the 4% FCM, suggesting that TTO may be beneficial in calves’ ability to acquire immunity and enhance body resistance.

Physiological and biochemical indicators in the blood can reflect the function and nutritional metabolism of the different organs and tissues of animals [14]. ALT and AST are relatively important transaminases in ruminants, and not only are they related to protein metabolism, but their activity levels can also reflect liver-related functions [15]. ALT and AST are important indicators reflecting the degree of liver damage and liver cell damage and can reflect the permeability of the hepatocyte membrane [16]. High doses of TTO could significantly reduce AST activity in the serum of weaned piglets, proving that TTO is beneficial in protecting the liver without causing liver damage [17]. Our study showed that the addition of 0.02% TTO tended to decrease AST activity in the dairy cows’ serum, indicating that TTO could improve liver function in postpartum dairy cows. ALP is widely distributed in the body’s liver, kidneys, bones, and other tissues. It is a key digestive and metabolic enzyme in the body and is involved in the metabolism of protein and fat in animals [18]. Studies have shown that ALP activity in serum is elevated when liver and gallbladder lesions develop [19]. The results of this study show that the addition of TTO had no significant effect on ALP activity, indicating that TTO did not cause damage to the liver and gallbladder of the dairy cows in the postpartum period.

Serum TP is composed of ALB and GLO, and a change in their content reflects the nutritional level of protein in the diet, the synthesis and digestion of protein by the animal organism, and the liver and kidney function [20]. ALB is synthesized in liver cells, and its activity can reflect liver function, the transport of nutrients, and the function of providing protein to the body. GLO is mainly composed of complement proteins and immunoglobulins, and changes in its content can reflect spleen function and immunity.

In this study, the addition of 0.02% TTO to the diet increased the content of GLO relative to the control, and the addition of 0.01% TTO to the TMR diet showed an increase in the content of ALB, indicating that the addition of TTO had no negative effect on the liver or spleen, and it may promote the immunity of dairy cows. CREA and BUN in serum can reflect the protein metabolism and amino acid balance of dairy cows [21]. Changes in CREA levels are related to glomerular filtration. When the kidneys are damaged, the excretion of CREA is hindered, resulting in increased serum CREA levels [22,23]. The BUN content in serum can reflect the protein level in the diet and protein catabolism in the body. In this study, the addition of TTO had no significant effect on the serum CREA and BUN contents in the postpartum dairy cows, indicating that TTO had no impact on the protein metabolism and kidneys of the dairy cows.

In postpartum, dairy cows often suffer from NEB due to decreased DMI and glucose, which can have a negative impact on the entire lactation cycle. The glucose level plays an important role in the normal function of the animal body and tissue and can reflect the body’s ability to balance the absorption, metabolism, and transport of sugar [24]. Glucose profiles are strongly linked to the short-term effect of the current energy and/or protein intake [25]. As an important energy source for dairy cows, glucose makes a difference in lactation and material turnover. The need for glucose was significantly increased in the postpartum period due to the onset of lactation. Mammary glucose uptake increased numerically, improving energy availability and inducing production responses [26]. In the present study, it was found that the 0.01% and 0.02% TTO groups in the TMR diets can contribute to the level of glucose in the postpartum dairy cows, indicating that the addition of TTO to TMR diets may have a positive effect on the lactation and nutritional metabolism in postpartum dairy cows. The serum levels of CHO, TG, HDL, and LDL reflect the absorption and metabolism of lipids by the animal organism [27]. The level of TG reflects the body’s blood lipids. When the TG content in the body is too high, the excess TG accumulates in the liver, increasing the burden on the liver and even leading to the occurrence of fatty liver [28]. In this study, the addition of TTO had no significant effect on the TG, CHO, HDL, and LDL levels.

The immunoglobulins in serum mainly include IgG, IgM, and IgA, which reflect the body’s immunity level. IgM is the earliest antibody that appears in the primary humoral immune response and can have anti-infection and virus-neutralizing effects by binding to antibodies to dissolve pathogens [29]. IgA has a better immune function in the local mucosa of the respiratory tract and digestive tract and infected membrane tissue [30]. IgG is the main antibody in serum, accounting for about 75% of immunoglobulins, and it has the function of binding antigens to activate, complement, and kill pathogens to resist infection effectively [31]. IgG is the main component of antibacterial, antitoxin, and antiviral antibodies and is also an important material that provides dairy cows protection against infection. Since IgG has the highest content and is also the only immunoglobulin that can cross the placental barrier, it plays an important role in the anti-infection of dairy cows. Adding resveratrol to the diet can increase the level of IgG in dairy cows [32]. In this experiment, the addition of TTO to the TMR diets had no profound effect on IgM and IgA concentrations. Remarkably, the addition of TTO to the TMR diets increased the level of serum IgG in the postpartum dairy cows, suggesting that the addition of TTO to TMR diets is beneficial to enhance immunity in postpartum dairy cows and that it has no adverse effects on the health of dairy cows.

## 5. Conclusions

The present study demonstrates that using TTO can improve lipid metabolism and enhance the immunity of postpartum dairy cows. TTO may be a novel resolution strategy for body condition recovery and the improvement of milk performance.

## Figures and Tables

**Table 1:** Ingredients and nutrient composition of the experimental diet (% of DM).

Item	%
Powder mix ^1^	22.73
Expanded soybean meal	2.19
Soybean hulls	2.19
Whole cottonseed	4.39
Alfalfa	3.95
Oats	3.29
Alfalfa silage	6.58
Alfalfa corn	48.25
Molasses	2.19
Yeast	0.07
Fat powder	0.66
Lysine	0.02
Sodium bicarbonate	0.64
Sodium chloride	1.21
Methionine	0.07
Urea	0.18
Vitamin and mineral mix ^2^	1.39
Nutrient composition	
NE_L_ ^3^ (MJ/kg)	6.78
CP	16.15
NDF	33.51
ADF	21.72
Ash	7.12
Ca	0.42
P	0.25
EE	3.75

^1^ Powder mix contained 70% corn and 30% soybean meal. ^2^ Vitamin and mineral mix contained 510 mg/kg Cu, 1380 mg/kg Zn, 2700 mg/kg Mn, 26 mg/kg Se, 170 mg/kg Fe, 20 mg/kg I, 4 mg/kg, 180 kIU/kg vitamin A, 45 kIU/kg vitamin D, and 1400 kIU/kg vitamin E. ^3^ Except for NE_L_, which is a calculated value, the rest are measured values.

**Table 2:** Effects of TTO on production performance in postpartum dairy cows.

Item	Treatment ^1^			SEM	*p-*Value
	Control	0.01% TTO	0.02% TTO		
DMI (kg/d)	15.21	16.76	15.14	0.33	0.07
Milk yield (kg/d)	27.16	28.45	28.89	0.37	0.13
Milk fat (%)	3.88	4.05	4.07	0.39	0.58
4% FCM (kg/d) ^2^	26.68	28.67	29.41	0.53	0.09
Feed efficiency ^3^	1.80	1.77	1.95	0.05	0.29

^1^ Twenty-four perinatal Holstein cows were fed TMR without TTO (as the control group), with 0.01% TTO or 0.02% TTO kg of DM basis. ^2^ 4% FCM (kg/d) = 0.4 × milk yield (kg/d) + 15 × milk yield (kg/d) × milk fat (%). ^3^ Feed efficiency = 4% FCM/DMI.

**Table 3:** Effects of TTO on serum-enzyme-related indices of bovine in postpartum period.

Item	Treatment ^1^			SEM	*p-*Value
	Control	0.01% TTO	0.02% TTO		
ALT	24.20	20.54	18.30	1.33	0.19
AST	92.29	85.16	81.54	1.93	0.063
ALP	60.38	55.25	57.63	1.37	0.32

^1^ Twenty-four perinatal Holstein cows were fed TMR without TTO (as the control group), with 0.01% TTO or 0.02% TTO kg of DM basis.

**Table 4:** Effects of TTO on serum-nitrogen-content-related indices of bovine in postpartum period.

Item	Treatment ^1^			SEM	*p-*Value
	Control	0.01% TTO	0.02% TTO		
TP (g/L)	71.55	72.54	73.21	0.56	0.50
ALB (g/L)	26.00	27.71	26.11	0.35	0.08
GLO (g/L)	45.55 ^b^	44.83 ^b^	47.10 ^a^	0.25	<0.05
CREA (μmol/L)	60.01	61.41	60.53	0.80	0.79
BUN (μmol/L)	5.20	5.07	4.91	0.13	0.67

^1^ Twenty-four perinatal Holstein cows were fed TMR without TTO (control group), with 0.01% TTO or 0.02% TTO kg of DM basis. ^a,b^ Means in the same row with different superscripts differ significantly for treatment effect.

**Table 5:** Effects of TTO on serum glucose and lipid metabolism in postpartum dairy cows.

Item	Treatment ^1^			SEM	*p-*Value
	Control	0.01% TTO	0.02% TTO		
Glucose (mmol/L)	2.79 ^b^	3.02 ^ab^	3.21 ^a^	0.06	<0.05
CHO (mmol/L)	2.84	2.77	2.80	0.06	0.93
TG (mmol/L)	0.152	0.139	0.133	0.005	0.233
HDL (mmol/L)	2.99	2.87	3.11	0.51	0.15
LDL (mmol/L)	1.67	1.65	1.39	0.06	0.14

^1^ Twenty-four perinatal Holstein cows were fed TMR without TTO (control group), with 0.01% TTO or 0.02% TTO kg of DM basis. ^a,b^ Means in the same row with different superscripts differ significantly for treatment effect.

**Table 6:** Effects of TTO on IgG, IgA, and IgM in postpartum dairy cows.

Item (g/L)	Treatment ^1^			SEM	*p-*Value
	Control	0.01% TTO	0.02% TTO		
IgG	5.20 ^b^	5.36 ^ab^	5.59 ^a^	0.07	0.045
IgM	2.38	2.43	2.89	0.12	0.18
IgA	0.41	0.42	0.43	0.007	0.40

^1^ Twenty-four perinatal Holstein cows were fed TMR without TTO (control group), with 0.01% TTO or 0.02% TTO kg of DM basis. ^a,b^ Means in the same row with different superscripts differ significantly for treatment effect.

## Data Availability

Not applicable.
